# Clinical characteristics of colonoscopy in 448 patients in the Zanzibar Archipelago: a cross-sectional study

**DOI:** 10.11604/pamj.2022.41.310.34185

**Published:** 2022-04-18

**Authors:** Li-Shuai Qu, Mariam Mohamed Gubi

**Affiliations:** 1 Digestive Endoscopy Center, Mnazi Mmoja Hospital, Stone Town, Zanzibar Archipelago,; 2 China Medical Team, Affiliated Hospital of Nantong University, Jiangsu, China

**Keywords:** Colonoscopy, colorectal cancer, cross-sectional study, Zanzibar

## Abstract

**Introduction:**

the aim was to investigate the demographic characteristics, primary colonoscopy findings, main indications, and feature of colorectal cancer (CRC) patients at Mnazi Mmoja Referral Hospital in the Zanzibar Archipelago, Tanzania.

**Methods:**

between December 2013 and October 2021, a total of 448 eligible participants were finally enrolled in present cross-sectional study. Demographic information and primary colonoscopy findings of each participant were retrieved.

**Results:**

among all enrolled subjects, 205 (45.80%) are females, remaining 243 (54.20%) are males. The median age of present cross-sectional study was 47 years old (ranging from 8 to 90 years). The main presenting indications included diarrhea (22.54%), abdominal pain (21.21%), hematochezia (18.53%), difficult defecation (16.96%), mucoid stool (10.49%), and anemia (8.70%). The common identified colonoscopy findings comprised colitis (28.57%), colonic polyps (25.22%), CRC (17.63%), inflammatory bowel disease (IBD) (13.52%), hemorrhoids (4.24%), and colonic diverticulum (4.02%), respectively. Unconditional logistic regression analyses demonstrated the elder group had significant higher risk of CRC (OR, 1.03; 95% CI, 1.01-1.03, P< 0.001), meanwhile a significant higher possibility of suffering hematochezia (OR, 2.29; 95% CI, 1.32-3.99, P=0.003) and anemia (OR, 2.96; 95% CI, 1.46-6.00, p= 0.003) in CRC group.

**Conclusion:**

the present study demonstrated that colitis, colonic polyps, CRC, and IBD are the most common colonoscopy diagnoses in Zanzibar. The indication of hematochezia or anemia showed a statistically higher risk of CRC.

## Introduction

Colonoscopy examination continues to be the most effective method to investigate the reason for lower gastrointestinal symptoms, such as diarrhea, abdominal pain, hematochezia, difficult defecation, or mucoid stool, and so on [1]. Currently, a large majority of colorectal cancer (CRC), inflammatory bowel disease (IBD), and colonic polyps were finally diagnosed by colonoscopy. The invention and progress of colonoscopy technology is a milestone in the modern gastroenterology [2,3]. Colonoscopy examination is an ideal procedure for identifying organic disorders of the lower digestive tract and it has been extensively accepted in not only diagnostic but also minimally invasive treatment [4].

The United Republic of Tanzania is a country in East Africa within the African Great Lakes region and is recognized as one of the poorest countries in the world. The Zanzibar archipelago, a semiautonomous region of Tanzania, includes Unguja, Pemba, and many other smaller islands on the Indian Ocean, with a total population of approximately 1.6 million [5]. Although colonoscopy examination has been proved to be highly effective and safe worldwide, this medical service was yet to be widely available in many developing regions including Zanzibar. As far as we know, no previous studies published to date yet discussed the clinical features and colonoscopy outcomes in Zanzibar. In addition, epidemiological data on CRC and potential warning symptoms are still lacking.

In present cross-sectional study within the past 9 years, we aimed to survey the demographic characteristics, primary colonoscopy findings, main indications, and the status of CRC patients underwent colonoscopy service in Zanzibar.

## Methods

**Study setting and design:** digestive endoscopy center in Mnazi Mmoja Referral Hospital in Stone Town, Unguja was established in December 2013, and it is the only hospital performing colonoscopy service in the whole Zanzibar. A cross-sectional study was conducted to evaluate the demographic characteristics, primary colonoscopy findings, main indications, and features of CRC patients in this national hospital.

**Study population:** the data was retrospectively retrieved from all medical in-patients and clinic out-patients who had received colonoscopy examination between December 2013 and October 2021in Mnazi Mmoja Referral Hospital.

**Selection criteria:** all subjects received the process of checking the name, gender, and ages of above recruited participants. No duplicate cases were enrolled into final analysis.

**Procedure of participant’s enrollment:** five hundred and fifty-seven (557) participants underwent colonoscopy were enrolled in this cross-sectional study. We had excluded 69 duplicate cases in this group, including 16 subjects with inability to tolerate during the procedure of colonoscopy examination, 27 participants with inadequate intestinal preparation and severe stool retention in rectum, and 26 cases underwent interventions. According to the medical record, 16 patients with inability to tolerate had all received repeat colonoscopy under sufficient sedation about two weeks later, 27 patients with inadequate intestinal preparation had been assigned second bowel preparation for colonoscopy and all of them had successfully received the colonoscopy. We also retrieved the record of 26 patients underwent interventions and all of them had accepted colonoscopy and histological evaluation before intervention in our unit. Consequently, a total of 448 eligible participants were finally enrolled into the analyses.

**Data collection:** information on recruited participants was extracted from electronic endoscopy reporting system, including the sex and age at the time of colonoscopy examination, the primary indications for colonoscopy, endoscopic diagnosis, and final pathological results.

**Variables:** key variables of this study included the number of enrolled subjects, gender, age, main indication and colonoscopy finding of each participant, and clinical feature of CRC patients.

**Ethical approval:** the present study was conducted according to the Helsinki Declaration and was approved by the research ethics committee at Mnazi Mmoja Referral Hospital in Stone Town, Unguja, Zanzibar Archipelago.

**Statistical analysis:** all data are presented as means ± SD, proportions, or median (range). To compare the values between the different groups, Fisher exact tests or Pearson´s Χ^2^ were conducted for categorical variables and the Student´s t-test was performed for continuous variables with normal distributions, respectively. Unconditional binary logistic regression model was used to estimate the odds ratios (ORs) of CRC related high risk factors and corresponding 95% confidence intervals (CIs). All statistical tests were two-tailed, and a P value of less than 0.05 was considered statistically significant. All statistical analyses were performed using the Statistical Program for Social Sciences (SPSS 23.0 for Windows; SPSS, Inc., Chicago, IL).

## Results

**Figure 1:**
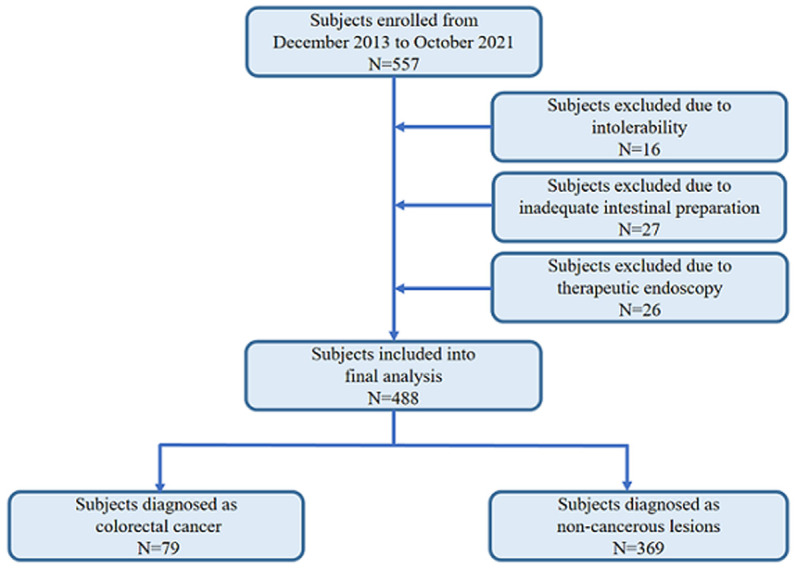
flow chart of participant’s inclusion and exclusion from this cross-sectional study

**Demographic characteristics of the study participants:** during the period of present retrospective observational study, a total of 448 participants underwent colonoscopy examination were recruited (Figure 1). Among above enrolled subjects, 205 (45.80%) are females, remaining 243 (54.20%) are males. The median age of this cross-sectional study was 47 years (ranging from 8 to 90 years). The 41 to 60 years age group had the highest frequency of 163 (36.38%) participants, followed by the 21 to 40 years age group with 146 (32.59%) patients, more than 60 years age group with 118 (26.34%) subjects, while less than 20 years age group had the lowest frequency of 21 (4.69%) cases, respectively. The details of sex and age distribution were shown in Table 1.

**Table 1:** patient characteristics and primary colonoscopy findings

Characteristics	Number (N)	Frequency (%)
No. of patients	448	
Male: female ratio	243: 205	54.20: 45.80
Age distribution (year)		
≤20	21	4.69%
21-40	146	32.59%
41-60	163	36.38%
>60	118	26.34%
**Colonoscopy findings**		
Colitis	128	28.57%
Colonic polyps	113	25.22%
Colorectal cancer	79	17.63%
Inflammatory bowel disease	61	13.62%
Normal findings	20	4.46%
Hemorrhoids	19	4.24%
Colonic diverticulum	18	4.02%
Sub mucosal tumor	3	0.67%
Colonic parasite	3	0.67%
Solitary rectum ulcer	3	0.67%
Rectum abscess	1	0.22%

**Year distribution of colonoscopy examinations:** from December 2013 to October 2021, 448 colonoscopy examinations distributed in nearly 9 years. The highest frequency of 98 participants appeared in 2018, followed by 87 subjects in 2019 and 63 patients in 2021, then 57 cases in 2014 and 49 cases in 2015, respectively. Compared with other years, the number of participants receiving colonoscopy examination was respectively 31 and 16 cases in 2015 and 2016 for the shortage of endoscopic consumables, only 45 patients received colonoscopy examination in 2020 because of the global pandemic of COVID-19.

**Primary colonoscopy findings:** of the 448 colonoscopies, 315 (70.31%) were complete, whereas the remaining 133 (29.69%) cases were considered as incomplete. Several factors were associated to incompletion: technical difficulties, patient intolerance, and the presence of severe bowel inflammation and obstructive lesions accounted for most of the incomplete colonoscopy examinations. In present data, 128 (28.57%) participants demonstrated colitis and this was the most popular identified colonoscopy finding, 113 (25.22%) of the 448 subjects had colonic polyps. Other positive colonoscopy findings included IBD in 61 (13.52%) patients, hemorrhoids in 19 (4.24%) patients, colonic diverticulum in 18 (4.02%) cases, respectively. Among the 61 participants with IBD, 38 (62.30%) were endoscopically diagnosed with ulcerative colitis (UC), 17 (27.87%) with Crohn’s disease (CD), and remaining six cases were diagnosed as IBD-unclassified. Histologically confirmed CRC was diagnosed in 79 (17.63%) patients. Less commonly reported findings were submucosal tumor seen in 3 (0.67%) cases, colonic parasite in 3 (0.67%) cases, solitary rectum ulcer in 3 (0.67%) patients, and rectum abscess in only 1 (0.22%) patient. While 20 (4.46%) participants had normal colonoscopy findings. The frequency of enrolled participants classified according to the colonoscopy findings was shown in Table 1.

**Primary indications for colonoscopy examination:** among all included participants, the main presenting symptoms included diarrhea (22.54%), abdominal pain (21.21%), hematochezia (18.53%), difficult defecation (16.96%), mucoid stool (10.49%), anemia (8.70%), and other less common symptoms in 1.65% of all study participants (Table 2). All above subjects presented at least 1 months after becoming aware of their warning symptoms.

**Table 2:** primary indications of colonoscopy (N=448)

Main indications for colonoscopy	Number (N)	Frequency (%)
Chronic diarrhea	101	22.54%
Abdominal pain	95	21.21%
Hematochezia	83	18.53%
Difficult defecation	76	16.96%
Mucoid stool	47	10.49%
Anemia	39	8.70%
Other symptoms	7	1.56%

**Association between clinical characteristics and the risk of CRC:** among 448 recruited participants, 79 (17.63%) patients suffered CRC. We also invest the site of the tumor. In our result, most tumors originated from the rectum in 29 (36.71%) patients and the sigmoid colon in 27 (34.18%) cases, while 11 (13.92%) cases locating in ascending colon, 7 (8.86%) cases locating in descending colon, and remaining 5 (6.33%) cases locating in transverse colon. We therefore attempted to explore the association between main clinical symptoms and the risk of CRC. In subgroup analysis, there was no statistically significant difference in gender distribution between CRC group and the participants with non-cancerous lesions (p =0.775). The average ages were 55.23 ±15.88 years in CRC group and 46.02±17.71 years in controls, respectively. The elder group had significant higher risk of CRC (OR, 1.03; 95% CI, 1.01-1.03, P< 0.001). As listed in Table 3, we also analyzed the role of main indications in predicting the possibility of CRC before colonoscopy examination. Compared with the controls, unconditional logistic regression analyses demonstrated a significant higher possibility of suffering hematochezia (OR, 2.29; 95% CI, 1.32-3.99, P=0.003) and anemia (OR, 2.96; 95% CI, 1.46-6.00, p= 0.003) in CRC group. Meanwhile, there were no statistically differences in the incidence of diarrhea, abdominal pain, difficult defecation, and mucoid stool between CRC group and non-cancerous lesions.

**Table 3:** association between clinical characteristics and colorectal cancer

Variable	Colorectal cancer N= 79 (%)	Controls N= 369 (%)	Odds ratio (95% CI)	P-value
Male: female ratio	44 (55.70):35 (44.30)	199 (53.93):170 (46.07)	1.07 (0.66-1.75)	0.775
Age (years), mean ± SD	55.23 ± 15.88	46.02 ± 17.71	1.03 (1.01-1.05)	<0.001
Main indication for colonoscopy				
Chronic diarrhea	13 (16.46)	88 (23.85)	0.63 (0.33-1.19)	0.156
Abdominal pain	15 (18.99)	80 (21.68)	0.85 (0.46-1.57)	0.595
Hematochezia	24 (30.38)	59 (15.99)	2.29 (1.32-3.99)	0.003
Difficult defecation	8 (10.12)	68 (18.43)	0.50 (0.23-1.09)	0.079
Mucoid stool	5 (6.33)	42 (11.38)	0.53 (0.20-1.38)	0.190
Anemia	14 (17.72)	25 (6.78)	2.96 (1.46-6.00)	0.003

## Discussion

As mentioned previously, colonoscopy examination is considered as a convenient and mini-invasive method that plays an important role in the detection of colonic disorders. Furthermore, distinct clinical features and outcomes of colonoscopy examination have also been depicted in different geographical areas of the world [6-8]. However, the data in Zanzibar Archipelago remain unknown. To the best of our knowledge, this study represents the first ever research on colonoscopy examination in Zanzibar, Tanzania. Our results documented the demographic characteristics, primary colonoscopy findings, main indications, and the status of CRC patients underwent colonoscopy service in a national hospital of Zanzibar.

In our study, present data indicated that participants between 20 and 60 years old made up about 70% of the enrolled group and the proportion of male are slightly more than female in this group. All included 448 cases were evenly distributed in approximate 9 years except relative low quantity in 2014, 2015, and 2020 due to the shortage of endoscopic consumables and the influence of COVID-19 pandemic. As mentioned previously, colonoscopy examination was considered as complete if endoscope reached the ileum, the caecum, or the anastomosis in subjects with surgical resection for tumor. A previous multicentric study in the United Kingdom had reported that the percentage of complete colonoscopies ranged between 56.9% and 76.9% according to above criteria [7]. However, some institutes have also recommended a completion rate of about 90% was considered as a quality marker of an Endoscopy unit [9]. The completion rate of our unit was ranging from 56% to 70% according to different statistical caliber. We speculated the potential reason for remaining incompletion participants might be attribute to common condition of severe bowel inflammation and obstructive lesions, which caused by relative high incidence of IBD and advanced stage CRC in current study. Similar to the results of previous studies, the most common diagnoses who underwent colonoscopy in this unit were colitis, colonic polyps, CRC, and IBD, in order. Meanwhile, due to strict policy about indications, the proportion of asymptomatic and routine physical examination participants underwent colonoscopy was very small. The negative colonoscopy finding was found in only 4.46% of the patients. This data was much lower than most past similar studies published in Nigeria, Tunis, and Ghana across Africa [10-12]. It has been reported that the occurrence of colonic polyps in Africans was generally lower, the incidence ranging from 5% to 10.3% from several studies conducted in Zimbabwe, Nigeria, and Kenya [13-15]. However, a meta-analysis revealed that the prevalence of colonic polyps in the United States was as high as nearly 30% [16]. In our unit, most colonoscopy cases were performed by experienced doctors from China Medical Team and we found about 25% of enrolled subjects suffered colonic polyps. About this issue, we suggested following possible reasons for the discrepancy: racial disparity, endoscopic equipment, and doctor's operating experience. Meanwhile, this relative high incidence of polyps might attribute to potential selection bias for the strict indications for colonoscopy in Zanzibar. Unfortunately, due to the shortage of endoscopic devices and economic hardship, the proportion of patients receiving colonoscopy therapy was very low in Zanzibar. IBD is increasingly considered as a worldwide disease burden in 21^st^ century [17]. However, little data about its epidemiology was known in Africa [18,19]. In Zanzibar, no relevant information was available previously. As indicated above, IBD was the fourth leading causes for subjects enrolled in this study who underwent colonoscopy. The UC-to-CD ratio in this study was slightly higher than 2, and this result was similar to the report in Western countries.

In the past decades, a significant growth in CRC incidence has been documented in Tanzania and incidence varies around the country [20,21]. As we known, CRC often originates from precancerous lesions such as adenomatous polyps. Theoretically, both adenomas and CRCs can be visualized under colonoscopy. A previous observation study in northern Tanzania had reported a low rate of colonoscopy in participants. Only 38% of CRC patients had ever received a colonoscopy procedure conducted for diagnostic or screening purposes [22]. In Zanzibar, only 79 CRC cases in the past nine years were detected by colonoscopy. Low colonoscopy rates can attribute to lack of alert to warning symptoms, scarcity of equipment and local experienced operators. Regarding risk of colonoscopy indications, we performed a comparison between 79 CRC patients and remaining 369 participants with non-cancerous lesions. Most of the screening guidelines used in Africa were introduced from western countries and the common recommended age for initial screening was average 50 years old [23-25]. From our data, elder participants also presented a significant high risk of developing CRC risk. Similar to the study performed by Sibomana *et al*. [26], those presenting alarm indications of hematochezia or anemia were significantly more likely to get a diagnosis of CRC. Specifically, a patient presenting with hematochezia and anemia was 2.29 and 2.96 times more likely to yield a CRC endoscopic finding compared to one without above alarm symptoms, respectively.

**Strengths and limitations:** to best of our knowledge, this is the first study discussed the clinical characteristics, indications, and outcomes of colonoscopy over a period of nearly 9 years in Zanzibar. The results of the present research are representative to local people. There are also several limitations that should be considered. First, present study was limited in lack of a gold standard about assessing the sensitivity and specificity of colonoscopy examination. The potential possibility of false negative and false positive results still exists. Second, we could not retrieve the life and clinical information of the recruited patients, the relationship between living styles and positive endoscopic findings could not be evaluated.

**Generalizability of information generated:** although above limitations exist, the paucity of data from Zanzibar in the previous literature still makes our report a useful contribution. As a cross-sectional study, it is hard to draw a firm conclusion and a well-designed, large-scale prospective study should be performed in future.

**Funding:** this research did not accept any specific grant from funding agencies in the commercial, public, or not-for-profit departments.

## Conclusion

In summary, the present study demonstrated that colitis, colonic polyps, CRC, and IBD are the most common colonoscopy diagnoses in Zanzibar. The indication of hematochezia or anemia showed a statistically higher risk of CRC.

### What is known about this topic

- Colonoscopy is yet to be widely available in many developing regions;
- Epidemiological data in CRC and potential warning symptoms are still lacking in Zanzibar.

### What this study adds

- The present study revealed that colitis, colonic polyps, CRC, and IBD are the most common colonoscopy diagnoses in Zanzibar;
- The indication of hematochezia or anemia showed a statistically higher risk of CRC.

## References

[1] Waye JD (1992). Colonoscopy. CA Cancer J Clin.

[2] Gkolfakis P, Tziatzios G, Dimitriadis GD, Triantafyllou K (2017). New endoscopes and add-on devices to improve colonoscopy performance. World J Gastroenterol.

[3] Ishaq S, Siau K, Harrison E, Tontini GE, Hoffman A, Gross S (2017). Technological advances for improving adenoma detection rates: The changing face of colonoscopy. Dig Liver Dis.

[4] Gomez V, Wallace MB (2014). Advances in diagnostic and therapeutic colonoscopy. Curr Opin Gastroenterol.

[5] Saleh F, Kitau J, Konradsen F, Mboera LEG, Schiøler KL (2021). Emerging epidemics: is the Zanzibar healthcare system ready to detect and respond to mosquito-borne viral diseases?. BMC Health Serv Res.

[6] Cremers MI, Marques-Vidal P (2006). Colonoscopies in Portuguese district hospitals: a multicentric transverse study. Dig Liver Dis.

[7] Bowles CJ, Leicester R, Romaya C, Swarbrick E, Williams CB, Epstein O (2004). A prospective study of colonoscopy practice in the UK today: are we adequately prepared for national colorectal cancer screening tomorrow?. Gut.

[8] Baker FA, Mari A, Nafrin S, Suki M, Ovadia B, Gal O (2019). Predictors and colonoscopy outcomes of inadequate bowel cleansing: a 10-year experience in 28,725 patients. Ann Gastroenterol.

[9] Gorard DA, McIntyre AS (2004). Completion rate to caecum as a quality measure of colonoscopy in a district general hospital. Colorectal Dis.

[10] Olokoba AB, Obateru OA, Bojuwoye MO, Olatoke SA, Bolarinwa OK, Olokoba LB (2013). Indications and findings at colonoscopy in Ilorin, Nigeria. Niger Med J.

[11] Houissa F, Kchir H, Bouzaidi S, Debbeche R, Trabelsi S, Moussa A (2011). Colonoscopy in elderly: feasibility, tolerance and indications: about 901 cases. Tunis Med.

[12] Dakubo J, Kumoji R, Naaeder S, Clegg-Lamptey Jn (2008). Endoscopic evaluation of the colorectum in patients presenting with haematochezia at korle-bu teaching hospital accra. Ghana Med J.

[13] Alatise OI, Arigbabu AO, Agbakwuru EA, Lawal OO, Ndububa DA, Ojo OS (2012). Spectrum of colonoscopy findings in Ile-Ife Nigeria. Niger Postgrad Med J.

[14] Kayamba V, Nicholls K, Morgan C, Kelly P (2018). A seven-year retrospective review of colonoscopy records from a single centre in Zambia. Malawi Med J.

[15] Ismaila BO, Misauno MA (2013). Gastrointestinal endoscopy in Nigeria--a prospective two year audit. Pan Afr Med J.

[16] Heitman SJ, Ronksley PE, Hilsden RJ, Manns BJ, Rostom A, Hemmelgarn BR (2009). Prevalence of adenomas and colorectal cancer in average risk individuals: a systematic review and meta-analysis. Clin Gastroenterol Hepatol.

[17] Kaplan GG (2015). The global burden of IBD: from 2015 to 2025. Nat Rev Gastroenterol Hepatol.

[18] Hodges P, Kelly P (2020). Inflammatory bowel disease in Africa: what is the current state of knowledge?. Int Health.

[19] Windsor JW, Kaplan GG (2019). Evolving Epidemiology of IBD. Curr Gastroenterol Rep.

[20] Katalambula LK, Ntwenya JE, Ngoma T, Buza J, Mpolya E, Mtumwa AH (2016). Pattern and Distribution of Colorectal Cancer in Tanzania: A Retrospective Chart Audit at Two National Hospitals. J Cancer Epidemiol.

[21] Parker RK, Ranketi SS, McNelly C, Ongondi M, Topazian HM, Dawsey SM (2019). Colorectal cancer is increasing in rural Kenya: challenges and perspectives. Gastrointest Endosc.

[22] Herman AM, Hawkins AT, Misso K, Issangya C, Tarmohamed M, Mremi A (2020). Colorectal Cancer in Northern Tanzania: Increasing Trends and Late Presentation Present Major Challenges. JCO Glob Oncol.

[23] Rex DK, Boland CR, Dominitz JA, Giardiello FM, Johnson DA, Kaltenbach T (2017). Colorectal Cancer Screening: Recommendations for Physicians and Patients from the U.S Multi-Society Task Force on Colorectal Cancer. Am J Gastroenterol.

[24] Force USPST, Bibbins-Domingo K, Grossman DC, Curry SJ, Davidson KW, Epling Jr JW (2016). Screening for Colorectal Cancer: US Preventive Services Task Force Recommendation Statement. JAMA.

[25] European Colorectal Cancer Screening Guidelines Working G, von Karsa L, Patnick J, Segnan N, Atkin W, Halloran S (2013). European guidelines for quality assurance in colorectal cancer screening and diagnosis: overview and introduction to the full supplement publication. Endoscopy.

[26] Sibomana I, Karenzi ID, Niyongombwa I, Dusabejambo V, Kiswezi A (2019). Lower gastrointestinal bleeding at a referral hospital in Kigali, Rwanda: Clinical, colonoscopic and pathologic profiles. East and Central African Journal of Surgery.

